# Myosteatosis as an independent predictor for all-cause and cardiac mortality in initial-dialysis patients: a multicenter, retrospective cohort study

**DOI:** 10.3389/fphys.2025.1687179

**Published:** 2025-11-26

**Authors:** Shi-Mei Hou, Min Li, Jing-Yuan Cao, Yao Wang, Min Yang, Li Tian, Yan-Hong Ni, Bi-Xia Yang, Li-Rong Hao, Jian-Bing Hao, Chun-Bo Zou, Shu-Yan Zhang, A-Feng Miao, Min Yang, Chun-Hong Hu, Li Yuan, Jing Zheng, Jing-Jie Xiao, Jian Xu, Bin Wang

**Affiliations:** 1 Department of Nephrology, The Third Affiliated Hospital of Soochow University, Changzhou, China; 2 Institute of Nephrology, Zhong Da Hospital, School of Medicine, Southeast University, Nanjing, Jiangsu, China; 3 Institute of Nephrology, Taizhou People’s Hospital, Taizhou, Jiangsu, China; 4 Institute of Nephrology, Yangzhou First People’s Hospital, Yangzhou, Jiangsu, China; 5 Department of Nephrology, Southern University of Science and Technology Hospital, Shenzhen, China; 6 School of Medicine, Nantong University, Nantong, Jiangsu, China; 7 Institute of Geriatrics, Zhong Da Hospital, School of Medicine, Southeast University, Nanjing, Jiangsu, China; 8 Covenant Health Palliative Institute, R416 St Marguerite Health Services Center, Edmonton, AB, Canada; 9 Department of intensive care unit, Geriatric Hospital of Nanjing Medical University, Nanjing, Jiangsu, China

**Keywords:** all-cause mortality, cardiac mortality, dialysis, myosteatosis, nomogram

## Abstract

**Background:**

Myosteatosis is associated with adverse prognosis in diseases. We aimed to establish thresholds for myosteatosis, assess its association with all-cause and cardiac mortality in initial dialysis patients, and construct a myosteatosis-based survival nomogram. assessed the predictive value of myosteatosis with all-cause and cardiac mortality in initial-dialysis patients, and constructed a myosteatosis-based survival nomogram.

**Methods:**

This multicentric retrospective study included 383 initial-dialysis patients (1/2014–12/2019). Endpoints include all-cause and cardiac mortality. Skeletal Muscle Index (SMI, cm^2^/m^2^) and Skeletal Muscle Density (SMD, Hounsfield Units [HU]) were measured at the third lumbar vertebra (L3) level by computed tomography (CT). Sex-specific SMI and SMD thresholds predicted all-cause mortality through the receiver operating characteristic (ROC) curves. The Cox models assessed myosteatosis-associated mortality risks. Survival prediction models were built using univariate and multivariate Cox proportional hazards regression in the training cohort, followed by internal and external validation.

**Results:**

Patients were predominantly aged 18–65 years (n = 298, 77.81%), with males comprising 60.84% (n = 233). All-cause mortality was 22.72% (n = 87), of which 52.87% (n = 46) were attributed to cardiac causes. Sex-specific SMD cutoffs for predicting all-cause mortality were 32.46 HU (AUC = 0.707) in males and 34.58 HU (AUC = 0.690) in females (both P < 0.05). Myosteatosis was associated with higher all-cause (36.7%) and cardiac mortality (19.8%) (both P < 0.001), and independently predicted both outcomes (all-cause mortality: HR = 3.203, 95% CI:1.937–5.296; cardiac mortality: HR = 3.418, 95% CI:1.718–6.802). The myosteatosis-based nomogram achieved a C-index of 0.761, validated in real-world data.

**Conclusion:**

Sex-specific myosteatosis thresholds (males: SMD ≤32.46 HU; females: ≤34.58 HU) derived from L3-CT independently predicted all-cause and cardiac mortality in initial-dialysis patients. The myosteatosis-based survival nomogram demonstrated moderate-to-good predictive accuracy and potential clinical utility.

## 1 Introduction

Chronic kidney disease (CKD) represents a significant global public health burden, associated with markedly increased mortality and adverse outcomes, particularly cardiovascular events. In addition to the traditional risk factors (Morel et al., 2022), recent studies have shown that sarcopenia is also a risk factor for increased cardiovascular events and mortality in patients with CKD recent studies have identified sarcopenia as a risk factor for mortality in CKD (Harada et al., 2017; Zhang et al., 2022). Beyond muscle loss, impaired muscle quality characterized by intramuscular fat infiltration, termed myosteatosis, has emerged as another important prognostic factor. Furthermore, CKD patients frequently experience impaired muscle quality characterized by excessive intramuscular fat infiltration, a condition termed myosteatosis.

Myosteatosis is characterized by abnormal distribution of adipose tissue between and within myocytes (Reinders et al., 2016). While its precise pathogenesis remains incompletely understood, contributing factors likely include ectopic fat accumulation driven by advanced age, sex steroid hormone deficiency, mitochondrial dysfunction, and prolonged immobility (Kim et al., 2020; Chang et al., 2014; Lowell and Shulman, 2005; Cree et al., 2010). Current studies demonstrates that myosteatosis independently predicts mortality in diverse clinical populations, including mechanically ventilated ICU patients (Looijaard et al., 2016), individuals with rectal cancer (Blauwhoff-Buskermolen et al., 2016; Brown et al., 2018; Xiao et al., 2020), and kidney transplant recipients (Morel et al., 2022). Moreover, studies indicate a significantly higher prevalence of myosteatosis in both peritoneal dialysis (PD) and hemodialysis (HD) patients compared to healthy controls (Lindholm et al., 1986; Johansen et al., 2003).

Superior to muscle biopsy, which is impracticable in a large population-based study because of its invasiveness, computed tomography (CT) imaging, a non-invasive tool, has been commonly used to evaluate the quantity and quality of skeletal muscle (Mourtzakis et al., 2008). Low attenuation of abdominal CT at the third lumbar vertebra (L3) provides surrogate markers for myosteatosis (Goodpaster et al., 2000). However, up to date, the diagnostic threshold for myosteatosis in end-stage kidney disease (ESKD) patients remain undefined, and the relationship between myosteatosis and mortality in initial dialysis patients has not been fully yielded. Therefore, this study aimed to establish optimal diagnostic thresholds for myosteatosis based on skeletal muscle density (SMD) measured by CT images at the L3 in initial dialysis patients and to evaluate the association of myosteatosis with all-cause and cardiac mortality within this cohort.

## 2 Materials and methods

### 2.1 Study design and population

This was a multicenter, retrospective study. Consecutive patients who were initially admitted to dialysis (a dialysis duration ≤3 months; hemodialysis frequency: 3 times per week, 4 h per session; peritoneal fluid daily exchange capacity: 8,000–10000 L) at Zhongda Hospital Affiliated to Southeast University, the First People’s Hospital Affiliated to Yangzhou University, Taizhou People’s Hospital Affiliated to Nanjing Medical University, and Changzhou First People’s Hospital Affiliated to Soochow University from January 2014 and December 2019, were screened.

The inclusion criteria for this study were patients aged 18–75 years. The exclusion criteria of the study were as follows: (1) malignancy or expected survival less than 3 months, (2) cirrhosis, (3) inflammatory bowel disease, (4) kidney transplantation, (5) Guillain-Barre syndrome, (6) Alzheimer’s disease, (7) amputation, and (8) inadequate unenhanced abdominal CT images within 1 month before or after initial dialysis. Ultimately, 1,236 subjects were excluded due to loss of follow-up or meet the exclusion criteria (Figure 2). Which was provided in Supplementary Methods. The study protocol was approved by the Ethics Committee of southeast university affiliated Zhongda Hospital, China (registration number: 2022ZDSYLL003-P01). The retrospective registration number was ChiCTR 2300068453. The date of registration was 2023–02–20. This observational study did not involve any intervention measures for patients and the risk to the subjects was not greater than the minimum risk. Given that, exemption of informed consent was applied for and approved. All methods were performed in accordance with the relevant guidelines and regulations.

### 2.2 Assessment of abdominal skeletal muscle area and density at L3 level

**FIGURE 1:**
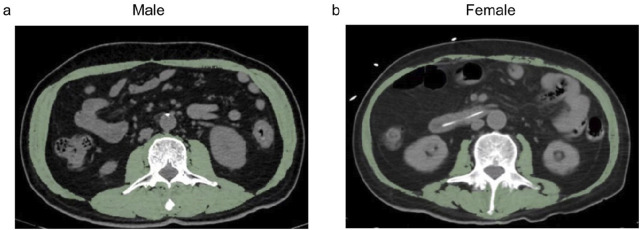
Representative images of L3 axial CT slices of skeletal muscle distribution in initial-dialysis patients. Both skeletal muscle area and density were measured in **(a)** male patients and **(b)** female patients.

Total skeletal muscle area (SMA) and SMD were evaluated using abdominal CT images at the L3 level (Figure 1 *)*. The skeletal muscle index (SMI, cm^2^/m^2^) was calculated as SMA divided by height squared. Details of image acquisition and processing were provided in the Supplementary Methods. Non-enhanced abdominal CT images with 5 mm thickness were obtained from all four centers. All CT examinations were performed using standardized protocols: 120 kV; automated dose modulation (auto-mA and smart-mA for GE Healthcare, CareDose 4D for Siemens Healthineers, and DoseRight for Philips); 512 × 512 matrix; and 0.625 mm collimation. A single axial slice at the L3 level was used to quantify total skeletal muscle area (SMA) and SMD (Figure 1). The skeletal muscle index (SMI, cm^2^/m^2^) was calculated as SMA divided by height squared. SMA and SMD were assessed at the mid-L3 plane by trained nephrologists, blinded to clinical and biological data. Muscles including erector spinae, quadratus lumborum, obliquus externus abdominis, obliquus internus abdominis, transverses abdominis and rectus abdominis were measured by the use of a Hounsfield unit (HU) threshold of −29 to +150. Measurement reliability was assessed in 40 randomly selected participants. Intraclass correlation coefficients (ICCs) were 0.998 for SMA and 0.995 for SMD, indicating excellent agreement. The image analyses were conducted with software ImageJ (NIH ImageJ version1.47, http://rsbweb.nih.gov/ij/).

### 2.3 Measurements of covariates

Clinical data, demographic data and laboratory results were collected according to the hospital electronic medical records. Details were provided in the Supplementary Methods.

### 2.4 Grouping

In this study, the subjects were divided into myosteatosis group and non-myosteatosis group based on the receiver operating characteristic (ROC) curve of sex-specific SMD and all-cause mortality. When constructing the nomogram, we further divided the patients into a training cohort and an independent external validation cohort according to different dialysis centers.

### 2.5 End points

All included patients were followed up from the date of dialysis initiation until lost to follow-up or death or the end of the 3-year follow-up, whichever occurred first. The endpoint in this study was all-cause and cardiac mortality. Details were provided in the Supplementary Methods. The time and cause of death were assessed by senior residents who were blinded to other results. Patients were followed up using a standardized protocol that included outpatient follow-up, electronic medical records of rehospitalization and telephone consultation. Cardiac mortality was defined as death due to any cardiovascular event other than non-cardiac vascular death such as stroke and peripheral vascular disease.

### 2.6 Statistical analysis

Continuous variables were expressed as mean ± standard deviation (SD) if they were normally distributed or as median (interquartile range, IQR) if they were skewed distributed. Categorical variables were presented as number (percentage). Comparison of clinical values between two groups were performed using Chi-square (χ^2^) test or Fisher’s exact test for categorical variables, Student’s t-test for normal distributions and Mann-Whitney U test for non-normally-distributed data. ROC curve analyses were used to determine the sensitivity and specificity for sex-specific skeletal muscle index and SMD in the identification of all-cause mortality. The area under the curve (AUC) and the 95% confidence interval (CI) were calculated. The optimal cut-off value of sex-specific SMD was derived from the maximum Youden index. Kaplan-Meier curves combined with log-rank test were used to compare the cumulative survival rate for myosteatosis with all-cause mortality and cardiac mortality. Univariate and multivariate Cox proportional hazards regression analysis was used to further explore the relationship between myosteatosis and all-cause mortality and cardiac mortality. We constructed four Cox proportional hazards regression models. The adjusted variables in different models were described in Supplementary Methods. All results were presented as Hazard Ratio (HR) and 95% CI.

We further divided the patients into a training cohort (n = 256) and an external validation cohort (n = 127). A dynamic nomogram and a web-based calculator of survival probability are constructed by using the model with the minimum Akaike Information Criterion AIC (AIC) to screen independent risk factors in multiple Cox proportional risk analysis. Internal verification of the prediction model by using bootstrapping methods. Internal validation of the nomogram was conducted using the bootstrap method with 1,000 resamples. To ensure the reproducibility of the bootstrap procedure, a random seed was set to 123. The concordance index (C-index) was calculated based on this process. The discrimination performance of the nomogram model for predicting survival probability was analyzed using concordance index (C-index), calibration curve, the Brier score, the Hosmer-Lemeshow goodness-of-fit test and time-dependent ROC curves. The clinical practicality of the nomogram was evaluated by decision curve analysis.

Two-tailed *P* values <0.05 was considered statistically significant. All analyses were performed using IBM SPSS Statistics for Windows, version 26 (IBM Corp, Armonk, NY, USA), GraphPad Prism 9 and R software (version 4.3.1; http://www.r-project.org).

## 3 Results

### 3.1 Basic characteristics

**FIGURE 2:**
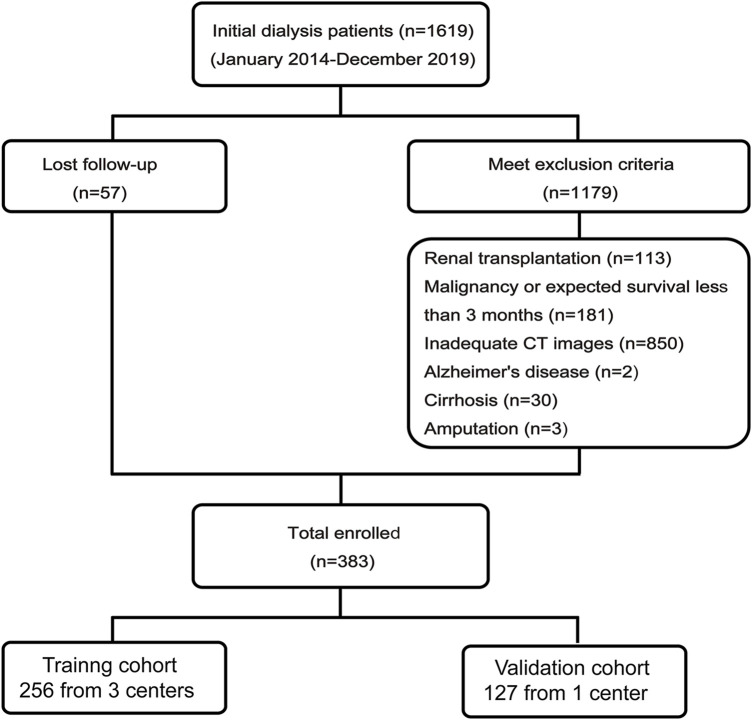
The enrollment flow chart.

Finally, a total of 383 patients who completed follow-up with complete baseline data were included in this study. Patients were divided into a training cohort consisting of 256 patients from three centers and an independent external validation cohort consisting of 127 patients from a separate center (Figure 2).

The study population was predominantly aged 18–65 years (n = 298; 77.81%) and mostly male (n = 233; 60.84%). HD was the primary dialysis modality (n = 314; 81.98%). During the 3-year follow-up period, the overall all-cause mortality was 22.72% (n = 87), of which 52.87% (n = 46) died from cardiac causes (Table 1).

**TABLE 1:** Baseline data according to myosteatosis defined by computed tomography images at L3 level.

Variables	Myosteatosis group (n = 177)	Non-Myosteatosis group (n = 206)	Whole cohort (n = 383)	P-value
Demographic and disease data				
Age (year), n (%)				<0.001
18-65 years	115 (64.97)	183 (88.84)	298 (77.81)	
66-75 years	62 (35.03)	23 (11.17)	85 (22.19)	
Sex, n (%)				<0.001
Male	84 (47.46)	149 (72.33)	233 (60.84)	
Female	93 (52.54)	57 (27.67)	150 (39.16)	
Smoking history, n (%)	42 (23.73)	55 (26.70)	97 (25.33)	0.505
Dialysis mode, n (%)				<0.001
Hemodialysis	164 (92.66)	150 (72.82)	314 (81.98)	
Peritoneal dialysis	13 (7.35)	56 (27.18)	69 (18.12)	
Comorbidities, n (%)				
Diabetes	87 (49.15)	77 (37.38)	164 (42.82)	0.020
Hypertension	158 (89.27)	190 (92.23)	348 (90.86)	0.315
Coronary heart disease	31 (17.51)	22 (10.68)	53 (13.84)	0.053
Medication history, n (%)				
Iron agent	97 (54.80)	121 (58.74)	218 (56.92)	0.438
EPO	155 (87.57)	194 (94.18)	349 (91.12)	0.023
compound α-keto acid tablets	86 (48.59)	112 (54.37)	198 (51.70)	0.259
Anthropometric data				
Heigh, m, Median (IQR)	1.65 (1.58 - 1.70)	1.70 (1.63 - 1.73)	1.67 (1.60 - 1.72)	<0.001
Weight, kg, Median (IQR)	63.00 (55.00 - 71.00)	65.00 (56.80 - 74.78)	64.70 (56.00 - 72.50)	0.207
BMI, kg/m2, Median (IQR)	23.66 (20.92 - 26.53)	23.39 (20.60 - 25.72)	23.44 (20.77 - 26.26)	0.318
CT data				
SMI, cm2/m2, Median (IQR)	45.77 (38.13 - 53.06)	45.77 (37.49 - 54.08)	45.88 (37.82 - 53.91)	0.848
Biological data				
Hb, g/l, Median (IQR)	81.00 (69.00 - 93.00)	86.00 (73.00 - 99.00)	83.00 (72.00 - 95.00)	0.028
PLT, ×109/l, Median (IQR)	178.00 (121.50 - 246.50)	167.00 (133.00 - 204758)	171.00 (129.00 - 219.00)	0.084
ALB, g/l, Mean ± SD	31.88 ± 6.43	34.49 ± 5.70	33.29 ± 6.18	<0.001
Scr/CysC, Median (IQR)	14.58 (12.30 - 16.88)	14.72 (12.53 - 17.89)	14.69 (12.38 - 17.47)	0.173
End points				
Cardiac mortality, n (%)	35 (19.77)	11 (5.34)	46 (12.01)	<0.001
All-cause mortality, n (%)	65 (36.72)	22 (10.68)	87 (22.72)	<0.001

Abbreviations: BMI, body mass index; EPO, erythropoietin; Hb, hemoglobin; PLT, platelet; ALB, albumin; SCr/CysC, serum creatinine divided by cystatin C; CT, computed tomography; SMI, skeletal muscle index; IQR, interquartile range; SD, standard deviation.

### 3.2 Comparison of clinical and laboratory characteristics between myosteatosis group and non-myosteatosis group

ROC curve analysis indicated that SMI lacked predictive value for all-cause mortality in both male (*P* = 0.518) and female patients (*P* = 0.203). In contrast, the optimal sex-specific SMD cutoff values for predicting all-cause mortality were 32.46 HU in males (sensitivity 64.7%, specificity 28%) and 34.58 HU in females (sensitivity 88.9%, specificity 53.5%). The AUC of sex-specific SMD was 0.707 (*P* < 0.001) in males and 0.690 (*P* = 0.001) in females (Supplementary Figure S1). Notably, SMD values were significantly higher in male patients compared to females (35.59 ± 8.76 HU vs. 30.30 ± 8.88 HU, *P* < 0.001). Based on these sex-specific SMD cutoff values (≤32.46 HU for males, ≤34.58 HU for females), 177 patients (46.2%) were classified into the myosteatosis group and 206 patients (53.8%) into the non-myosteatosis group.

As shown in Table 1, patients with myosteatosis were more likely to be older (aged 66–75 years), female, and receiving HD, and had a higher prevalence of diabete. Compared to the non-myosteatosis group, the myosteatosis group exhibited a lower proportion of erythropoietin use, shorter height, and lower levels of hemoglobin (Hb) and albumin (ALB) (all *P* < 0.05). Furthermore, both all-cause and cardiac mortality rates were significantly higher in the myosteatosis group (all *P* < 0.001). No other significant differences were observed between the groups.

### 3.3 Comparison of clinical and laboratory characteristics between training cohort and validation cohort

As shown in Table 2, the training cohort included 141 patients with myosteatosis and 115 without, while the validation cohort comprised 65 patients with myosteatosis and 62 without. Patients in the training cohort had a significantly higher prevalence of smoking history (*P* = 0.011), diabetes (*P* = 0.013) and coronary heart disease (*P* = 0.039). Iron agent use was also significantly more common in the training cohort (*P* < 0.001). Additionally, platelet levels and SMI were significantly higher in the training cohort (*P* < 0.001 and *P* = 0.033, respectively), while ALB levels were significantly lower (*P* = 0.012) compared to the validation cohort. No other significant differences in clinical characteristics were observed between the cohorts.

**TABLE 2:** Comparison of clinical and laboratory characteristics between the training cohort and the validation cohort.

Variables	Training cohort (n = 256)	Validation cohort (n = 127)	P-value
Demographic and disease data			
Age (year), n (%)			0.471
18-65 years	198 (77.34)	100 (78.74)	
66-75 years	58 (22.66)	27 (21.26)	
Sex, n (%)			0.164
Male	162 (63.28)	71 (55.91)	
Female	94 (36.72)	56 (44.09)	
Smoking history, n (%)	75 (29.30)	22 (17.32)	0.011
Dialysis mode, n (%)			0.084
Hemodialysis	216 (84.38)	98 (77.17)	
Peritoneal dialysis	40 (15.62)	29 (22.83)	
Comorbidities, n (%)			
Diabetes	121 (47.27)	43 (33.86)	0.013
Hypertension	234 (91.41)	114 (89.76)	0.599
Coronary heart disease	42 (16.41)	11 (8.66)	0.039
Medication history, n (%)			
Iron agent	161 (62.89)	57 (44.88)	<0.001
EPO	235 (91.80)	114 (89.76)	0.510
compound α-keto acid tablets	140 (54.69)	58 (45.67)	0.096
Anthropometric data			
Heigh, m, Median (IQR)	1.67 (1.60 - 1.72)	1.67 (1.60 - 1.72)	0.795
Weight, kg, Median (IQR)	65.00 (56.48 - 73.25)	62.00 (55.00 - 71.50)	0.251
BMI, kg/m2, Median (IQR)	23.66 (20.88 - 26.41)	23.36 (20.76 - 25.33)	0.289
Biological data			
Hb, g/l, Median (IQR)	84.00 (74.00 - 96.00)	82.00 (68.50 - 94.50)	0.248
PLT, ×109/l, Median (IQR)	183.50 (141.00 - 235.00)	146.00 (114.50 - 189.00)	<0.001
ALB, g/l, Mean ± SD	32.73 ± 6.29	34.40 ± 5.80	0.012
Scr/CysC, Median (IQR)	14.02 (12.43 - 17.05)	15.62 (12.33 - 18.01)	0.182
CT data			
Myosteatosis, n (%)	115 (44.92)	62 (48.82)	0.471
SMI, cm2/m2, Median (IQR)	46.44 (38.41 - 54.12)	43.92 (36.97 - 51.44)	0.033
End points			
All-cause mortality, n (%)	60 (23.44)	27 (21.26)	0.632

Abbreviations: BMI, body mass index; EPO, erythropoietin; Hb, hemoglobin; PLT, platelet; ALB, albumin; SCr/CysC, serum creatinine divided by cystatin C; CT, computed tomography; SMI, skeletal muscle index; IQR, interquartile range; SD, standard.

### 3.4 Predictive value of myosteatosis for all-cause and cardiac mortality in initial dialysis ESKD patients

**FIGURE 3:**
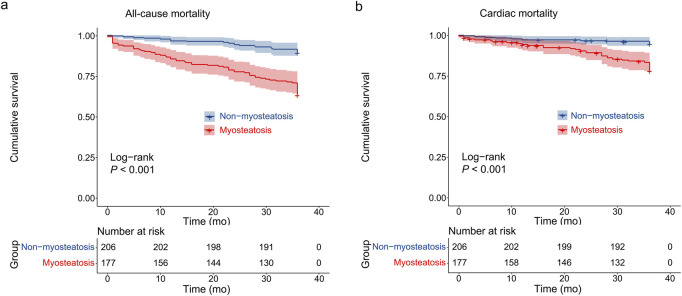
Kaplan - Meier survival curves. **(a)** The all-cause mortality and **(b)** cardiac mortality between myosteatosis and non-myosteatosis group were compared.

Kaplan-Meier analysis revealed significantly higher all-cause mortality and cardiac mortality rates in the myosteatosis group compared to the non-myosteatosis group (*P* < 0.001 for both; Figure 3).

Variables showing significant differences between groups in univariate analyses were included in multivariate Cox proportional hazards models. The events-per-variable ratio in multivariate models were detailed in Supplementary Tables S1,S2. Myosteatosis remained an independent predictor for both all-cause (HR = 3.195, 95% CI: 1.938–5.268, *P* < 0.001) and cardiac mortality (HR = 3.418, 95% CI: 1.718–6.802, *P* < 0.001). The performance of our model on the validation set was comparable to that on the test set, indicating no significant overfitting issue. Restricted cubic spline analysis within the Cox models demonstrated a linear association between sex-specific SMD and all-cause mortality (Supplementary Figure S2). Furthermore, we assessed the independent predictive value of myosteatosis for all-cause and cardiac mortality in four multivariate Cox regression analysis which were adjusted for confounding factors. As shown in Model 4, even after adjusting for serum albumin and hemoglobin in the fully adjusted Cox model, myosteatosis remained a significant predictor of all-cause mortality (HR: 3.203, 95% CI: 1.937–5.296, *P* < 0.001) and cardiac mortality (HR: 3.418, 95% CI: 1.718–6.802, *P* < 0.001) compared to those without myosteatosis (Table 3).

**TABLE 3:** The independent predictive value of myosteatosis for all-cause and cardiac mortality.

Model	All cause mortality		Cardiac mortality	
	HR (95% CI)	P-value	HR (95% CI)	P-value
Model 1^a^	4.023 (2.480, 6.526)	<0.001	4.333 (2.200, 8.535)	<0.001
Model 2^b^	3.524 (2.137, 5.812)	<0.001	3.756 (1.863, 7.571)	<0.001
Model 3^c^	3.304 (1.999, 5.462)	<0.001	3.418 (1.718, 6.802)	<0.001
Model 4^d^	3.203 (1.937, 5.296)	<0.001	3.418 (1.718, 6.802)	<0.001

^a^ Unadjusted.

^b^ Adjusted for BMI, SMI, age, and sex.

^c^ Adjusted for BMI, SMI, age, sex, dialysis mode, smoking history, comorbidities (diabetes, hypertension and coronary heart disease) and medications (iron agent, erythropoietin and compound α-keto acid tablets).

^d^ Adjusted for BMI, SMI, age, sex, dialysis mode, smoking history, comorbidities (diabetes, hypertension and coronary heart disease), medications (iron agent, erythropoietin and compound α-keto acid tablets) and laboratory results (Hb, PLT, ALB, and SCr/CysC).

Abbreviations: BMI, body mass index; SMI, skeletal muscle index; Hb, hemoglobin; PLT, platelet; ALB, albumin; SCr/CysC, serum creatinine divided by cystatin C; HR, hazard ratio; CI, confidence interval.

### 3.5 A nomogram for predicting overall survival in initial dialysis patients with ESKD

#### 3.5.1 Nomogram characteristics screening

Based on univariate Cox regression analysis results in the training cohort (Supplementary Table S3), multivariate Cox analysis identified advanced age (*P* = 0.038), diabetes (*P* < 0.001), and myosteatosis (*P* = 0.001) as independent predictors of all-cause mortality (Supplementary Figure S3).

#### 3.5.2 Nomogram construction and validation

**FIGURE 4:**
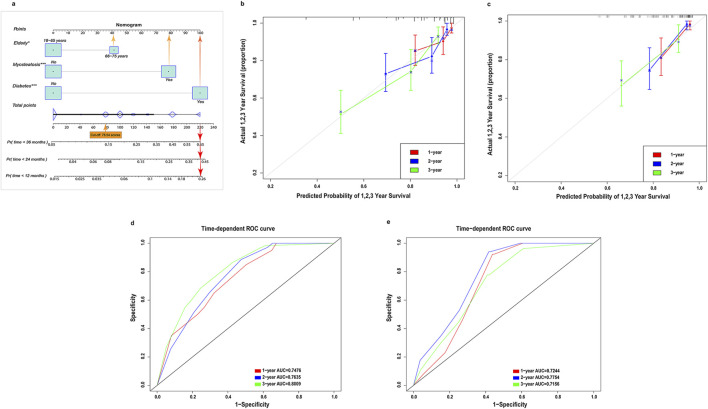
Construction of myosteatosis-based nomograms in initial-dialysis patients. **(a)** Myosteatosis-based nomograms of overall survival. The calibration curves for predicting 1-, 2-, and 3-year survival in training set **(b)** and in validation set **(c)**. The X-axis presents the predicted probability and the Y-axis shows the actual probability. **(d)** The ROC curves of nomogram for predicting the 1-, 2-, and 3-year overall survival rate in the training set (n = 256). **(e)** The ROC curves of nomogram for predicting the 1-, 2-, and 3-year overall survival rate in validation set (n = 127). Abbreviations: ROC: receiver operating characteristic.

Using these multivariate Cox regression results, we developed a dynamic, myosteatosis-based nomogram to predict 1-, 2-, and 3-year overall survival probabilities in initial dialysis patients. Based on the value of each variable, a certain score was obtained in the first line. The scores for each variable were then added together to obtain a total point, based on which the probability of 1,2,3-year risk of death was predicted for each patient. For example, the 1-, 2-, and 3-year risk of death for 70-year-old initial dialysis patient with ESKD, diabetes and myosteatosis was 25% (95% CI:0.640–0.870), 42% (95% CI:0.460–0.750), and 65% (95% CI:0.229–0.540), respectively (Figure 4a). Using the Kaplan-Meier-derived cutoff of 78.54 points, patients were stratified into high-risk and low-risk groups. To facilitate clinical use, we created a web-based calculator implementing this model. [Overall Survival Prediction Model. URL: https://houshimei141421.shinyapps.io/hsm141421/(accessed January 24, 2024)]. The operational workflow was as follows: first, SMD was obtained from routine abdominal CT imaging, and myosteatosis was classified using sex-specific thresholds. The clinician then entered the patient’s age, diabetes status, and myosteatosis category into the online tool, which generated an estimated survival probability.

The C-index for overall survival prediction in training cohort and in validation cohort was 0.761 (95%CI, 0.708–0.814)and 0.703 (95%CI, 0.623–0.783), respectively. The calibration curves for the probability of 1-, 2-, and 3-year overall survival demonstrated optimal consistency between the prediction by nomogram and actual observation both in both cohorts (Figures 4b,c). In the training cohort, the Brier scores were 0.08, 0.12, and 0.14 at 1, 2, and 3 years, respectively, indicating low prediction error. The Hosmer-Lemeshow tests were all non-significant (all *P* > 0.05), demonstrating excellent calibration. Similarly, in the validation cohort, the Brier scores remained low (0.09, 0.13, and 0.15 at 1, 2, and 3 years, respectively), and the Hosmer-Lemeshow tests showed no significant lack of fit (all *P* > 0.05), underscoring the robustness and generalizability of the nomogram.

**FIGURE 5:**
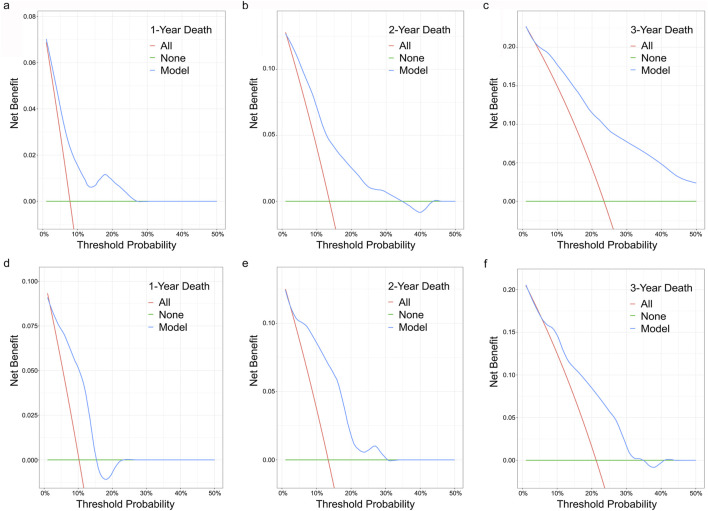
DCA for the proposed nomogram model. **(a–c)** DCA for the proposed nomogram model to predict 1-, 2-, and 3-year all-cause mortality in the training set. **(d–f)** DCA for the proposed nomogram model to predict 1-, 2-, and 3-year all-cause mortality in the validation set. Abbreviations: DCA: decision curve analyses.

To examine the discriminative ability of the nomogram, the ROC curve was plotted and the AUC was calculated. The AUC values for the nomogram to predict the 1-, 2-, and 3-year survival in the training cohort were 0.748, 0.764 and 0.801, while the values in the validation cohort were 0.724, 0.775 and 0.716, respectively (Figures 4d,e). Decision curve analysis (DCA) confirmed the clinical utility of the nomogram for predicting 1-, 2-, and 3-year all-cause mortality in both cohorts (Figure 5).

To evaluate the incremental predictive value of myosteatosis, we compared the full nomogram with a baseline model including only the traditional risk factors of age and diabetes. As shown in Supplementary Table S4, the full model demonstrated a significantly higher C-index than the baseline model in both the training cohort (0.761 vs. 0.698, P < 0.01) and the validation cohort (0.703 vs. 0.657, P < 0.05). It also demonstrated consistently lower Brier scores across all time points, indicating superior predictive accuracy. These findings confirm that myosteatosis adds meaningful and statistically significant prognostic value beyond traditional risk factors.

## 4 Discussion

This multicenter retrospective study of 383 initial dialysis patients with ESKD evaluated the prevalence and clinical impact of myosteatosis, determined by CT at the L3 level. Our key findings are threefold. First, we established optimal diagnostic cut-off values for myosteatosis using the ROC curve analysis based on sex-specific SMD at L3. Second, myosteatosis was highly prevalent in this population and emerged as an independent predictor of both all-cause and cardiac mortality. Third, we developed and validated the first dynamic nomogram incorporating traditional prognostic factors and myosteatosis to predict overall survival using real-world data. Additionally, we created a user-friendly web-based calculator to facilitate clinical application of this survival prediction model.

Myosteatosis, as characterized by the presence of intermuscular and intramuscular fat infiltration (Miljkovic and Zmuda, 2010), was confirmed to be related to ESRD. Lindholm et al. demonstrated twice the prevalence of biopsy-confirmed myosteatosis in PD patients compared to healthy controls (Lindholm et al., 1986). Keddar et al. further confirmed that myosteatosis severity correlates with declining residual diuresis and deteriorating renal function in PD patients (Keddar et al., 2020). Similarly, myosteatosis assessed by magnetic resonance imaging was significantly higher in HD patients than age- and sex-matched controls (Johansen et al., 2003). However, there is still no established diagnostic threshold for myosteatosis, especially in the population with CKD. A recent study reported that the cut-off points for myosteatosis was a mean SMD <41 HU for patients with a BMI <24.9 kg/m^2^ and a mean SMD <33 HU for patients with a BMI ≥25 kg/m^2^ in the liver transplant cohort (Czigany et al., 2021). While kidney allograft recipients defined myosteatosis as SMD below the 2.5th percentile of healthy populations (Morel et al., 2022). Keddar et al. defined a SMD below two SDs of young healthy persons as myosteatosis in patients with kidney failure (Keddar et al., 2020). These discrepancies underscore the critical need for standardized diagnostic guidelines specific to the ESKD population.

Currently, CT is recognized as a gold standard method for muscular fat quantification due to its non-invasive nature, safety, and widespread availability. Accordingly, our study utilized sex-specific SMD measurements from L3-level CT images to diagnose myosteatosis. We established optimal mortality-based diagnostic thresholds of 32.46 HU for males and 34.58 HU for females in initial dialysis ESKD patients. Notably, in contrast to our results, Xiao et al. showed a higher cut-off value for myosteatosis of 35.5 HU in male than that in female (32.5 HU) patients with colorectal cancer (Xiao et al., 2018), which was consistent with the study in the Netherlanders between 20 and 82 years old eligible for kidney donation, showing that the sex specific cut-off value for myosteatosis was 29.3 HU in male and 22.0 HU in female, respectively (van der Werf et al., 2018). The discrepancy may be due to differences in race and measurement software, highlighting the need to standardize instrumentation for the analysis of myosteatosis. Moreover, our findings confirmed a higher SMD in male compared to female, which was in accordance with several previous studies (Morel et al., 2022; Xiao et al., 2018; Zamboni et al., 2019). This phenomenon may be account for differences in muscle fiber phenotypes, muscle synthesis and catabolic pathways, sex steroid hormones and mitochondrial content (Avesani et al., 2023). It is important to note that the low specificity (28%-53.5%) at the selected diagnostic threshold reflects our study design, which prioritized maximizing sensitivity (64.7%-88.9%) for early screening. For myosteatosis, we consider the consequences of false negatives to outweigh those of false positives, as timely identification is critical to enable early clinical intervention and improve long-term prognosis.

Using our sex-specific thresholds, myosteatosis prevalence was 46% overall, which was similar to the value in non-metastatic colorectal cancer patients (Malietzis et al., 2016) and liver transplant recipients (Czigany et al., 2020), but higher than that in a population of kidney allograft transplantation^1^. Moreover, the prevalence of myosteatosis was 39% in younger patients (18–65 years) and 73% in elderly patients (66–75 years). Numerous studies confirmed that the prevalence of myosteatosis increased with age (Vedder et al., 2020; Miljkovic et al., 2016; Correa-de-Araujo et al., 2020), which is likely due to redistribution of adipose tissue (Addison et al., 2014) and muscle disuse, or physical inactivity (Tuttle et al., 2011). In this study, myosteatosis was more prevalent in hemodialysis (HD) than peritoneal dialysis (PD) patients. There may be the following reasons: Firstly, for the patients who enter maintenance dialysis initially, most patients preferentially choose PD to preserve the residual renal function. Second, compared with HD, PD patients have a higher quality of life, greater activity, better nutrition, a lower incidence of comorbidities, and higher muscle function and quality. Third, patients with MHD are more likely to have excessive fat accumulation in their muscles under the risk factors of prolonged immobility, severe anemia, and cardiovascular disease. These findings highlight the need for prioritized myosteatosis screening in elderly and HD populations, with prompt intervention where indicated.

In the present study, myosteatosis was associated with significantly increased 3-year all-cause mortality and cardiac mortality, serving as an independent predictor across four multivariable models. Recent studies yielded the same result in patients suffering from HD (Yajima, 2022), cancer (Dolan et al., 2019), liver transplantation (Czigany et al., 2021), renal transplantation (Morel et al., 2022), mechanical ventilation in intensive care (Looijaard et al., 2016), and even in healthy people without cardiovascular disease (Larsen et al., 2020). However, contrasting these results, one study found an association between intramuscular fat and 4-year mortality only in males aged 70–79 years, not in the overall cohort (Perkisas et al., 2018). Since the factors associated with myosteatosis such as obesity, insulin resistance and low-grade inflammation were also the risk factors leading to cardiovascular disease, there was no surprising that myosteatosis was associated with increased cardiac mortality in our study, which was consistent with a previous study in older men (Zhao et al., 2016). Furthermore, myosteatosis was related to cardiovascular events in PD patients (Keddar et al., 2020) and higher coronary artery calcification in young adults (Terry et al., 2017). Paradoxically, a recent study of renal transplant patients found no correlation between myosteatosis and cardiac mortality (Morel et al., 2022). The limited number of events in that cohort may explain this lack of association.

Importantly, we developed an effective nomogram integrating traditional clinical prognostic variables (age and diabetes mellitus) with myosteatosis to enable individualized assessment of overall survival in initial dialysis patients. An accessible web-based calculator facilitates the clinical implementation of this predictive model. Notably, our nomogram confirmed that myosteatosis is essential for predicting poorer outcomes, and its high C-index demonstrates its feasibility and reliability as a prognostic tool for identifying initial dialysis patients requiring more intensive intervention. Additional strengths include the external validation of our findings and the use of real-world data.

However, there are several limitations warrant consideration. As a retrospective cohort study with a relatively small sample size and short follow-up period, it is inherently unable to establish causation. Second, our study found that the prevalence rate of myosteatosis in HD patients was significantly higher than that in PD patients. Nevertheless, the exact influence of different dialysis mode on myosteatosis has not been clarified, highlighting further stratified analysis. Third, we could not analyze the relationship between dynamic changes in SMD and adverse outcomes. Fourth, By excluding patients without an abdominal CT scan performed within 1 month of dialysis initiation, we may have introduced selection bias, as these individuals were likely to have a higher burden of myosteatosis. Consequently, the true prevalence of myosteatosis in routine clinical practice may be underestimated. patients without abdominal CT within 1 month before or after initial dialysis were excluded in our study. However, these patients may have been accompanied by myosteatosis, leading to selection bias; Moreover, our findings cannot be extrapolated to individuals who did not undergo abdominal CT. Fifth, there was no data in our study on inflammation, insulin sensitivity, physical activity, dietary habit that might be related to myosteatosis, resulting in confounding bias. In our study, we did not directly assess key factors associated with myosteatosis, such as inflammation, nutritional status, insulin sensitivity, physical activity, and dietary habits. Although we adjusted for available clinical variables, the absence of precise measurements for these determinants may still have introduced residual confounding, potentially biasing the estimated association between myosteatosis and mortality. Sixth, although the results of the ROC analysis were statistically significant, the sensitivity and specificity were relatively low. Therefore, the optimal cutoff for SMD should be interpreted carefully, and the results may not be generalizable to other groups of patients. Finally, the nomogram based on myosteatosis constructed in this study was not compared with the one based on traditional risk factors. In future studies, we will further expand the multi-center collaboration to constantly test or modify the prediction model in clinical practice. Meanwhile, we will further validate the nomogram based on myosteatosis with more external data.

## 5 Conclusion

In summary, we have demonstrated a high prevalence of myosteatosis—diagnosed using sex-specific L3 CT SMD values—in initial dialysis patients with end-stage kidney disease (ESKD). Critically, myosteatosis was identified as an independent predictor of both all-cause and cardiac mortality. Most significantly, we are the first to develop and validate a nomogram that integrates this novel biomarker with established clinical predictors (age and diabetes) to provide personalized prediction of overall survival in this population.

## Data Availability

The raw data supporting the conclusions of this article will be made available by the authors, without undue reservation.

## Abbreviations

ALB, albumin; AUC AIC, area under the curve akaike information criterion; BMI, body mass index; CI, confidence interval; C-index, concordance index; CKD, chronic kidney disease; cm^2^, square centimetre; CT, computed tomography; CVD, cardiovascular disease; DCA, decision curve analysis; DM, diabetes mellitus; EPO, erythropoietin; ESKD, end-stage kidney disease; Hb, hemoglobin; HD, hemodialysis; HR, hazard ratio; ICU, intensive care unit; IQR, interquartile range; KT, kidney transplantation; L3, the third lumbar; MRI, magnetic resonance imaging; PLT, platelet; PD, peritoneal dialysis; ROC, receiver operating characteristic; SCr/CysC, serum creatinine divided by cystatin C; SD, standard deviation; SMA, skeletal muscle area; SMD, skeletal muscle density; SMI, skeletal muscle index.
