# Supplementary material for: Myosteatosis as an independent predictor for all-cause and cardiac mortality in initial-dialysis patients: a multicenter, retrospective cohort study
Source: Front Physiol. 2025 Nov 26;16:1687179. doi: 10.3389/fphys.2025.1687179 (PMC12689326; doi:10.3389/fphys.2025.1687179)
Supplement: Supplementary file 1 [file DataSheet1.pdf]

Supplementary Information

Supplementary Methods

Table S1 Univariate and multivariate Cox regression analysis of all-cause mortality in initial dialysis patients

Table S2 Univariate and multivariate Cox regression analysis of cardiac mortality in initial dialysis patients

Table S3 Univariate and multivariate Cox regression analysis of all-cause mortality in the training cohort

Figure S1 ROC curves of sex-specific SMD and SMI in predicting all-cause mortality in initial dialysis patients. (a) For male patients, the AUC of SMD was 0.707 and the cutoff value was 32.46 HU with a sensitivity of 65% and a specificity of 28% ($P < 0.001$). (b) For female patients, the AUC of SMD was 0.690 and the cutoff value was 34.58 HU with a sensitivity of 89% and a specificity of 54% ($P = 0.001$). (c-d) There was no significant association between SMI and all-cause mortality neither in male patients nor in female patients ($P > 0.05$). Abbreviations: ROC: receiver operating characteristic; SMD: skeletal muscle density; SMI: skeletal muscle index; AUC: area under the curve.

Figure S2 Dose-response relationship between sex-specific SMD and all-cause mortality. The ordinate is the HR, and the abscissa is the SMD. Lower sex specific SMD was associated with higher all-cause mortality. Abbreviations: SMD: skeletal muscle density; HR: hazard ratio.

Figure S3 The forest plot for risk factors of all-cause mortality in the training set. Abbreviations: ALB: albumin; rf: reference.

STROBE checklist

Supplementary Methods:

Study design and population

The exclusion criteria of the study were as follows: (1) malignancy or expected survival less than 3 months, (2) cirrhosis, (3) inflammatory bowel disease, (4) kidney transplantation, (5) Guillain-Barre syndrome, (6) Alzheimer's disease, (7) amputation, and (8) inadequate unenhanced abdominal CT images within 1 month before or after initial dialysis.

Assessment of abdominal skeletal muscle area and density at L3 level

Non-enhanced abdominal CT images with 5mm thickness were obtained from all the four centers. All CT examinations were performed with the following parameters: 120 kV; automated dose modulation using automA and smartmA for GE Healthcare machines, CareDose 4D for Siemens Healthineers, and DoseRight for Philips; matrix 512 × 512; collimation of 0.625 mm. Single-slice transverse CT images at L3 was determined to total skeletal muscle area (SMA) and SMD.

SMA and SMD at the central plane of the L3 level were evaluated by trained nephrologists, blinded to clinical and biological data. Tissues including erector spinae, quadratus lumborum, obliquus externus abdominis, obliquus internus abdominis, transverses abdominis and rectus abdominis were measured by the use of Hounsfield unit (HU) threshold –29 to + 150 for muscle. The intraclass correlation coefficient (ICC) was used to evaluate the consistency of the measurements from 40 randomly selected patients. The ICCs of 0.998 for SMA, 0.995 for skeletal muscle radiodensity. The image analyses were conducted with software Image J (NIH Image J version1.47, http://rsbweb.nih.gov/ij/).

Measurements of covariates

Clinical data, demographic data and laboratory results included age, sex, smoking history, dialysis mode, Complications prior to initial entry into maintenance dialysis such as diabetes mellitus (DM), hypertension and coronary heart disease (CHD), Medications prior to initial entry into maintenance dialysis (iron agent, erythropoietin and compound α-keto acid tablets), height, weight, hemoglobin (HB), platelet count (PLT), predialysis albumin (ALB), predialysis serum creatinine (SCr) and cystatin C. Body mass index (BMI) was calculated using the formula: BMI = weight (kg) / height squared (m^2). Estimated glomerular filtration rate (eGFR) was calculated using the CKD-EPI formula as follow: eGFR=141× (SCr/K) ^a× (0.993) ^age×1.080 (For females, K = 0.7, if SCr ≤ 0.7 mg/dl, a = -0.329, if SCr > 0.7 mg/dl, a = -1.209; For males, K = 0.9, if SCr ≤ 0.9 mg/dl, a = -0.411, if SCr > 0.9 mg/dl, a = -1.209) [S1].

End points

The time and cause of death were assessed by senior residents who were blinded to other results. Patients were followed up using a standardized protocol that included outpatient follow-up, electronic medical records of rehospitalization and telephone consultation. Cardiac mortality was defined as death due to any cardiovascular event other than non-cardiac vascular death such as stroke and peripheral vascular disease.

Statistical analysis

The adjusted variables in four Cox proportional hazards regression models were (a) Unadjusted; (b) Adjusted for BMI, age and sex; (c) Adjusted for BMI, age, sex, dialysis mode, smoking history, comorbidities and medications; (d) Adjusted for BMI, age, sex, dialysis mode, smoking history, comorbidities, medications and laboratory results, respectively.

Supplementary references:

S1. National Clinical Guideline Centre (UK). Chronic Kidney Disease (Partial Update): Early

Identification and Management of Chronic Kidney Disease in Adults in Primary and Secondary Care. London: National Institute for Health and Care Excellence (UK); July 2014.

Supplementary Tables:

Table S1 Univariate and multivariate Cox regression analysis of all-cause mortality in initial dialysis patients

Variables	Univariate Cox regression analyses		Multivariate Cox regression analyses	
	HR（95%CI）	*P*-Value	HR（95%CI）	*P*-Value
Myosteatosis	4.023 (2.480-6.526)	<0.001	3.195 (1.938-5.268)	<0.001
Sex (Male)	0.877 (0.572-1.344)	0.547		
Age (66-75 years)	2.442 (1.583-3.767)	<0.001	1.606 (1.027-2.510)	0.038
Dialysis methods (Hemodialysis)	2.400 (1.160-4.967)	0.018		
Smoking history	1.456 (0.929-2.283)	0.102		
Diabetes	3.175 (2.024-4.981)	<0.001	2.701 (1.717-4.247)	<0.001
Hypertension	0.917 (0.443-1.898)	0.815		
Coronary heart disease	2.374 (1.452-3.882)	0.001		
Iron agent	0.970 (0.635-1.482)	0.889		
EPO	0.922 (0.446-1.908)	0.827		
Compound α - keto acid tablets	1.075 (0.705-1.638)	0.738		
BMI, kg/m^2	0.979 (0.933-1.027)	0.377		
Hb, g/l	0.998 (0.988-1.009)	0.758		
PLT, ×10^9/l	1.002 (1.000-1.004)	0.027		
ALB, g/l	0.962 (0.930-0.995)	0.025		
Scr/CysC	1.008 (0.975-1.041)	0.641		
SMI, cm^2/m^2	1.010 (0.994-1.028)	0.226		

Abbreviations: BMI, body mass index; EPO, erythropoietin; Hb, hemoglobin; PLT, platelet; ALB, albumin; SCr/CysC, serum creatinine divided by cystatin C; SMI, skeletal muscle index; HR, hazard ratio; CI, confidence interval.

Table S2 Univariate and multivariate Cox regression analysis of cardiac mortality in initial dialysis patients

Variables	Univariate Cox regression analyses		Multivariate Cox regression analyses	
	HR（95%CI）	*P*-Value	HR（95%CI）	*P*-Value
Myosteatosis	4.333 (2.200-8.535)	<0.001	3.418 (1.718-6.802)	<0.001
Sex (Male)	1.056 (0.581-1.923)	0.857		
Age (66-75 years)	2.565 (1.418-4.639)	0.002		
Dialysis methods (Hemodialysis)	3.469 (1.076-11.182)	0.037		
Smoking history	1.637 (0.892-3.004)	0.111		
Diabetes	4.236 (2.192-8.183)	<0.001	3.133 (1.588-6.182)	0.001
Hypertension	1.320 (0.410-4.256)	0.642		
Coronary heart disease	3.612 (1.948-6.697)	<0.001	2.275 (1.202-4.308)	0.012
Iron agent	1.567 (0.846 2.902)	0.154		
EPO	0.996 (0.357-2.777)	0.994		
Compound α - keto acid tablets	1.197 (0.668- 2.144)	0.546		
BMI, kg/m^2	1.010 (0.947-1.077)	0.756		
Hb, g/l	1.001 (0.986-1.015)	0.945		
PLT, ×10^9/l	1.003 (1.000- 1.005)	0.076		
ALB, g/l	0.969 (0.925-1.015)	0.184		
Scr/CysC	1.017 (0.975-1.060)	0.431		
SMI, cm^2/m^2	1.018 (0.996-1.042)	0.112		

Abbreviations: BMI, body mass index; EPO, erythropoietin; Hb, hemoglobin; PLT, platelet; ALB, albumin; SCr/CysC, serum creatinine divided by cystatin C; SMI, skeletal muscle index; HR, hazard ratio; CI, confidence interval.

Table S3 Univariate and multivariate Cox regression analysis of all-cause mortality in the training cohort

Variables	Univariate Cox regression analyses		Multivariate Cox regression analyses	
	HR（95%CI）	*P*-Value	HR（95%CI）	*P*-Value
Myosteatosis	3.884 (2.191-6.885)	<0.001	2.756 (1.515-5.013)	0.001
Sex (Male)	0.834 (0.498-1.398)	0.491		
Age (66-75 years)	2.840 (1.699-4.747)	<0.001	1.769 (1.033-3.031)	0.038
Dialysis methods (Hemodialysis)	2.271 (0.909-5.672)	0.079		
Smoking history	1.449 (0.857-2.450)	0.166		
Diabetes	4.773 (2.581-8.826)	<0.001	3.647 (1.928-6.899)	<0.001
Hypertension	0.943 (0.377-2.356)	0.900		
Coronary heart disease	1.944 (1.083-3.490)	0.026		
Iron agent	1.216 (0.711-2.079)	0.476		
EPO	0.944 (0.378-2.359)	0.902		
Compound α - keto acid tablets	0.976 (0.587-1.623)	0.925		
BMI, kg/m^2	0.981 (0.928-1.037)	0.495		
Hb, g/l	0.992 (0.978-1.005)	0.236		
PLT, $\times 10^9$/l	1.002 (1.000-1.004)	0.097		
ALB, g/l	0.955 (0.918-0.993)	0.022		
Scr/CysC	1.006 (0.969-1.045)	0.742		
SMI, cm^2/m^2	1.013 (0.993-1.033)	0.203		

Abbreviations: BMI, body mass index; EPO, erythropoietin; Hb, hemoglobin; PLT, platelet; ALB, albumin; SCr/CysC, serum creatinine divided by cystatin C; SMI, skeletal muscle index; HR, hazard ratio; CI, confidence interval.

Supplementary Figures:

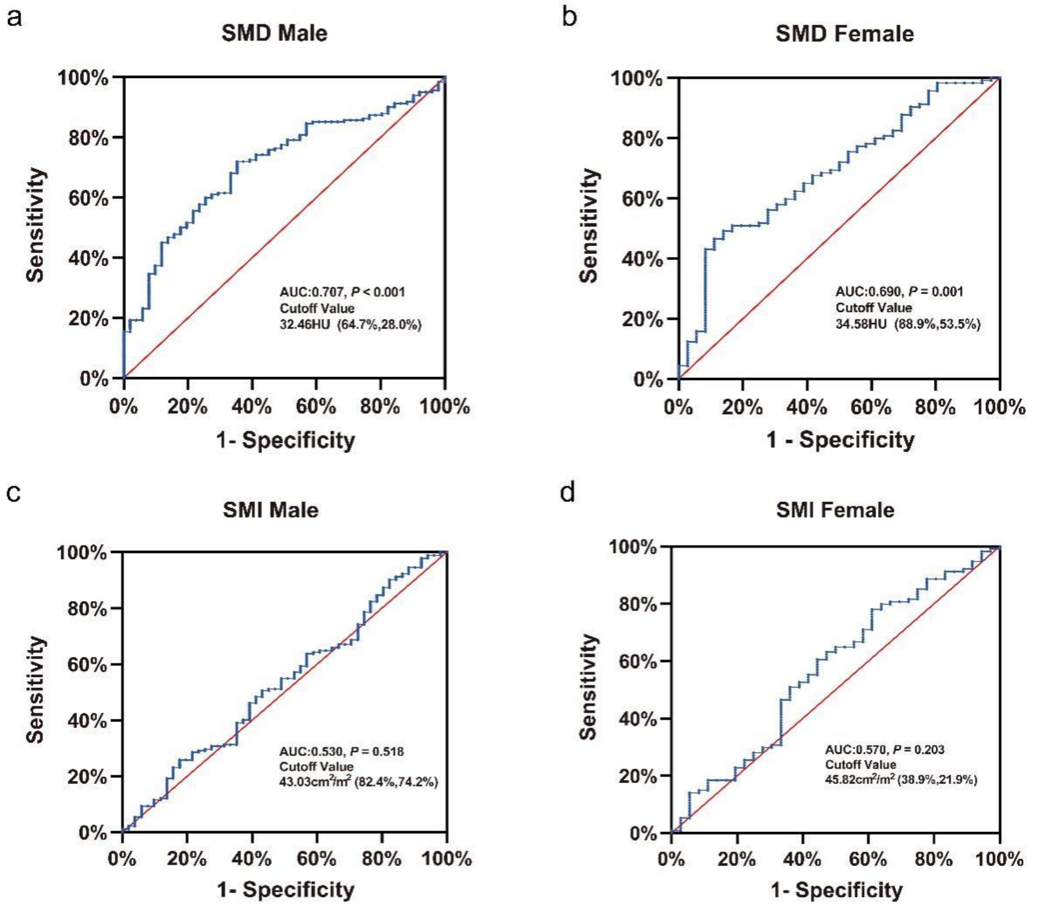

Figure S1 The ROC curves of the sex-specific SMD and SMI evaluated at the L3 CT level in predicting all-cause mortality in initial dialysis patients. (a) For male patients, the AUC of SMD was 0.707 and the cutoff value was 32.46 HU with a sensitivity of 64.7% and a specificity of 28.0% (P < 0.001). (b) For female patients, the AUC of SMD was 0.690 and the cutoff value was 34.58 HU with a sensitivity of 88.9% and a specificity of 53.5% (P = 0.001). (c-d) There was no significant

association between SMI and all-cause mortality neither in male patients nor in female patients (P > 0.05). Abbreviations: ROC: receiver operating characteristic; SMD: skeletal muscle density; SMI: skeletal muscle index; AUC: area under the curve.

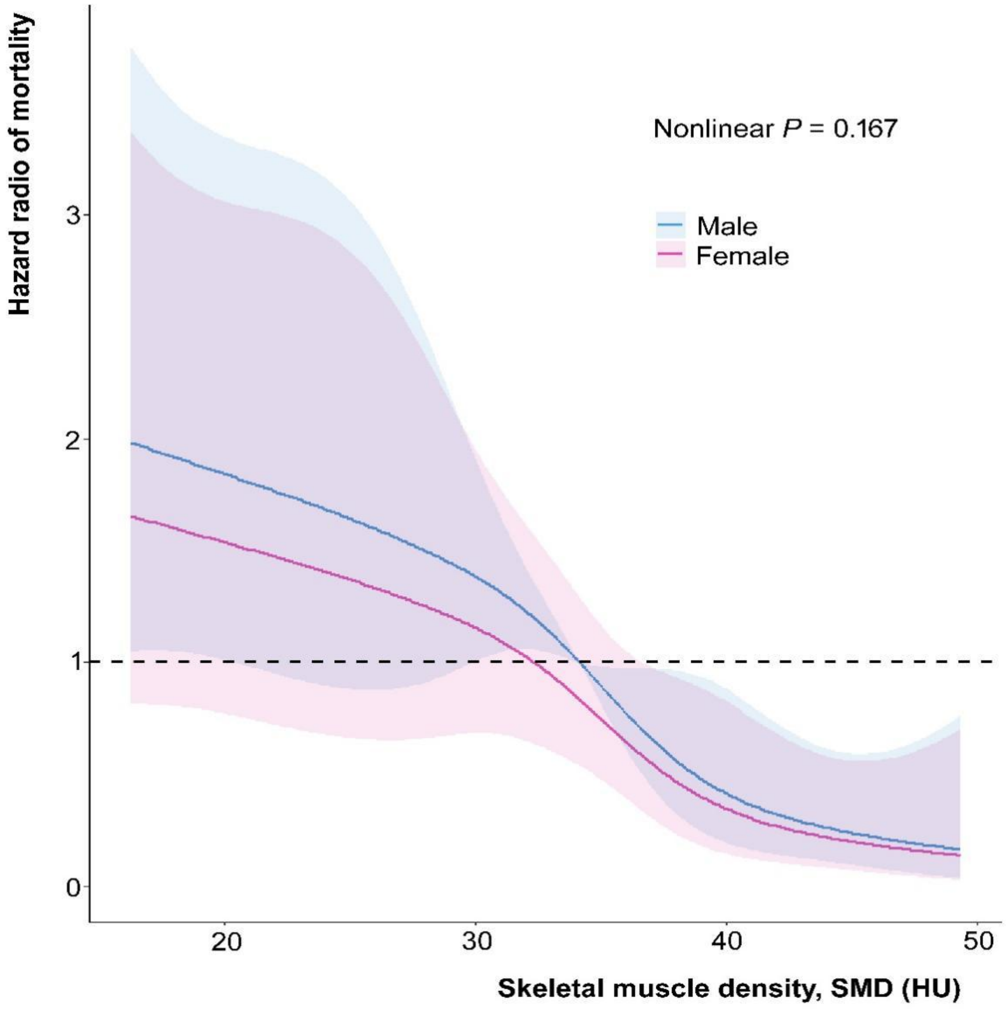

Figure S2 Dose-response relationship between sex-specific SMD and all-cause mortality. The ordinate is the HR, and the abscissa is the SMD. Lower sex specific SMD was associated with higher all-cause mortality. Abbreviations: SMD: skeletal muscle density; HR: hazard ratio.

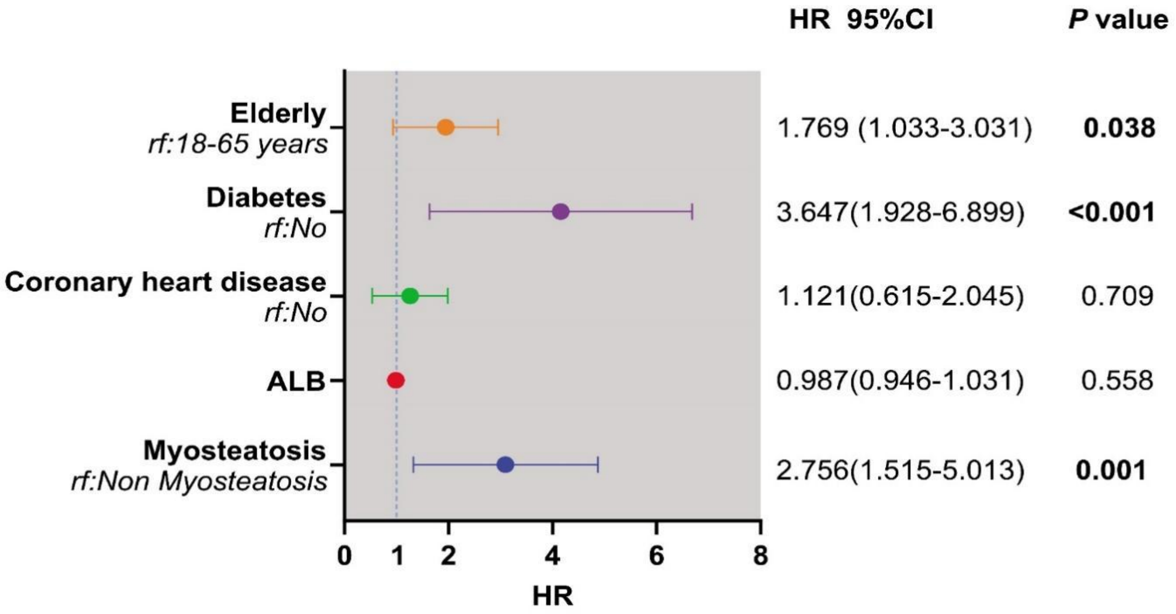

Figure S3 The forest plot for risk factors of all-cause mortality in the training set. Abbreviations: ALB: albumin; rf: reference.

STROBE Statement—Checklist of items that should be included in reports of ***cohort studies***

	Item No	Recommendation	Page No
**Title and abstract**	1	(*a*) Indicate the study's design with a commonly used term in the title or the abstract<br>(*b*) Provide in the abstract an informative and balanced summary of what was done and what was found	1,3,4
**Introduction**			
Background/rationale	2	Explain the scientific background and rationale for the investigation being reported	5
Objectives	3	State specific objectives, including any prespecified hypotheses	5
**Methods**			
Study design	4	Present key elements of study design early in the paper	6

Setting	5	Describe the setting, locations, and relevant dates, including periods of recruitment, exposure, follow-up, and data collection	6,7
Participants	6	(*a*) Give the eligibility criteria, and the sources and methods of selection of participants. Describe methods of follow-up<br>(*b*) For matched studies, give matching criteria and number of exposed and unexposed	6,<br>Supplementary data
Variables	7	Clearly define all outcomes, exposures, predictors, potential confounders, and effect modifiers. Give diagnostic criteria, if applicable	6, Supplementary data
Data sources/ measurement	8*	For each variable of interest, give sources of data and details of methods of assessment (measurement). Describe comparability of assessment methods if there is more than	6, Supplementary data

		one group	
Bias	9	Describe any efforts to address potential sources of bias	6
Study size	10	Explain how the study size was arrived at	6
Quantitative variables	11	Explain how quantitative variables were handled in the analyses. If applicable, describe which groupings were chosen and why	7
Statistical methods	12	(*a*) Describe all statistical methods, including those used to control for confounding<br><br>(*b*) Describe any methods used to examine subgroups and interactions<br>(*c*) Explain how missing data were addressed<br>(*d*) If applicable, explain how loss to follow-up was addressed<br>(*e*) Describe any sensitivity analyses	7, Supplementary Data

Results			
Participants	13*	(a) Report numbers of individuals at each stage of study—eg numbers potentially eligible, examined for eligibility, confirmed eligible, included in the study, completing follow-up, and analysed<br>(b) Give reasons for non-participation at each stage<br>(c) Consider use of a flow diagram	8
Descriptive data	14*	(a) Give characteristics of study participants (eg demographic, clinical, social) and information on exposures and potential confounders<br>(b) Indicate number of participants with missing data for each variable of interest<br>(c) Summarise follow-up time (eg, average and total amount)	8-9

Outcome data	15*	Report numbers of outcome events or summary measures over time	9-11

Main results	16	(*a*) Give unadjusted estimates and, if applicable, confounder-adjusted estimates and their precision (eg, 95% confidence interval). Make clear which confounders were adjusted for and why they were included<br>(*b*) Report category boundaries when continuous variables were categorized<br>(*c*) If relevant, consider translating estimates of relative risk into absolute risk for a meaningful time period	9-11
Other analyses	17	Report other analyses done—eg analyses of subgroups and interactions, and sensitivity analyses	9,10
**Discussion**			
Key results	18	Summarise key results with reference to study objectives	11
Limitations	19	Discuss limitations of the study, taking into account sources of potential bias or imprecision. Discuss both direction and	14

		magnitude of any potential bias	
Interpretation	20	Give a cautious overall interpretation of results considering objectives, limitations, multiplicity of analyses, results from similar studies, and other relevant evidence	12,13
Generalisability	21	Discuss the generalisability (external validity) of the study results	13
**Other information**			
Funding	22	Give the source of funding and the role of the funders for the present study and, if applicable, for the original study on which the present article is based	18,19

*Give information separately for exposed and unexposed groups.

Note: An Explanation and Elaboration article discusses each checklist item and gives methodological background and published examples of transparent reporting. The STROBE checklist is best used in conjunction with this article (freely available on the Web sites of PLoS Medicine at http://www.plosmedicine.org/, Annals of Internal Medicine at http://www.annals.org/, and Epidemiology at http://www.epidem.com/). Information on the STROBE Initiative is available at http://www.strobe-statement.org.
